# Bidirectional Reflectometry. Part II. Bibliography on Scattering by Reflection from Surfaces

**DOI:** 10.6028/jres.080A.022

**Published:** 1976-04-01

**Authors:** Joseph C. Richmond, Jack J. Hsia

**Affiliations:** Institute for Basic Standards, National Bureau of Standards, Washington, D.C. 20234

**Keywords:** Bibliography, emittance, heat transfer, measurement techniques, periodic surfaces, polarization, random surfaces, reflectance, reflectance of coherent radiation, scattering, scattering theory, surface roughness, transmittance

## Abstract

In connection with the work on development of a high resolution laser source bidirectional reflectometer, a large number of papers were collected dealing with various aspects of the geometrical distribution of the radiant energy reflected from surfaces of different types.

Each paper has been classified into one or more classes on the basis of its technical content. There are eight general classes, with several subclasses in some of the general classes.

Because of the interest in this field, the bibliography is being published as a service to the public.

A large number (371) of papers was collected, dealing with various aspects of the geometrical distribution of the radiant energy scattered by reflection from surfaces, and particularly from rough surfaces. These papers were referred to during the development of a high-resolution laser-source bidirectional reflectometer.

Each paper is classified in the Bibliography into one or more of thirteen subject categories on the basis of its technical content. A key to the symbols used in the bibliography is given in table 1.

In addition, each paper by number has been classified in a second system, consisting of eight primary classes, with several subclasses in several of the primary classes, in table 2.

The bibliography, while extensive, is by no means complete. Few additions have been made since about 1972. However, it is sufficiently extensive to be of significant help to workers in the field, and particularly to those just beginning work in the field.

The references from archival journals should be available in any good technical library. Most of the reports that have not been published in the archival literature are available from the National Technical Information Service. Most of these are identified by an AD number, or other identifying number by which they can be ordered.

Copies of all papers listed are on file in the Radiometric Physics Section of the National Bureau of Standards, and may be read at NBS if not otherwise available.

**Table 1:** Subject Categories

Symbol	
R	Reflectance
Em	Emittance
T	Transmittance
S	Scattering
P	Polarization
Ma	Mathematics
H	Heat Transfer Between Rough Surfaces
Ex	Experimental
Th	Theoretical
Su	Survey
L	Laser
SM	Surface Roughness Measurement
Ap	Apparatus

**Table 2:** Primary Classes

**NOMENCLATURE AND APPLICATION**
8, 42, 50, 52, 97, 101, 102, 113, 125, 132, 141, 143, 144, 154, 155, 180, 195, 216, 237, 238, 243, 275, 314, 315, 327, 341, 345
**SURFACE ROUGHNESS MEASUREMENT**
3, 12, 13, 16, 36, 61, 64, 65, 66, 69, 73, 80, 87, 95, 98, 99, 111, 115, 127, 133, 135, 136, 149, 157, 162, 171, 176, 190, 226–232, 245, 247, 255, 257, 258, 262, 265, 267, 277, 281, 286, 308, 312, 331, 347, 359, 361–365
**THEORY (RANDOM DISTRIBUTION SURFACE ROUGHNESS)**
SMALL FACETS SURFACE ASSUMPTION
18–21, 41, 77, 137, 138a, 181, 198, 210, 217, 223, 241, 261, 280, 292, 306, 321, 326, 328, 330
KIRCHHOFF-HUYGENS SOLUTION
9, 11, 32, 34, 36, 54, 81, 82, 90, 91, 92, 114, 116, 121, 137, 140, 150, 153, 160, 166, 178, 179, 254, 263, 264, 268, 299, 304, 311
OTHERS
10, 23, 24, 25, 32, 53, 67, 70, 76, 79, 84, 88, 89, 96, 105, 108, 109, 112, 119, 120, 123, 126, 128, 134, 146, 159, 170, 183, 184, 185, 193, 194, 199, 202, 205, 211, 223, 233, 242, 244, 274, 291, 301, 303, 307, 313, 328, 339, 342, 343
**THEORY (PERIODIC DISTRIBUTION SURFACE ROUGHNESS)**
CONTINUOUS SURFACES (SINUSOIDAL, GRATING, V-GROOVE, etc.)
1, 5, 6, 7, 14, 17, 22, 29, 32, 49, 51, 55, 59, 60, 114, 142, 156, 161, 167, 168, 169, 188, 209, 213, 214, 215, 219, 233, 234, 284, 353, 367, 370
SPHERES AND DISTRIBUTION
37–40, 43, 44, 56, 57, 104, 158, 177, 187, 207, 246, 248, 272, 332, 334, 335, 337, 352, 355, 356, 366
CYLINDERS AND DISTRIBUTION
56, 68, 110, 147, 164, 174, 220, 222, 239, 250, 289, 293, 294, 332, 333, 336, 338, 340, 349, 350, 351
**SHADOWING AND POLARIZATION**
28, 30, 57, 62, 74, 103, 117, 118, 119, 122, 124, 129, 138a, 202, 203, 204, 210, 224, 225, 252, 253, 260, 261, 279, 287, 292, 298, 317, 348, 367, 368
**EXPERIMENTAL DATA (ON RANDOM DISTRIBUTION SURFACES)**
COHERENT SOURCE
15, 75, 84, 85, 131, 189, 191, 192, 194, 208, 283
INCOHERENT SOURCE
4, 26, 27, 33, 35, 45–48, 58, 70, 72, 73, 93, 102, 106, 130, 139, 145, 151, 163, 165, 212, 218, 221, 240, 251, 256, 259, 261, 270, 271, 273, 276, 278, 285, 295, 296, 297, 300, 309, 310, 316–318, 320, 323, 324, 325, 329, 346, 354, 357
**EXPERIMENTAL DATA (ON PERIODIC DISTRIBUTION SURFACES)**
78, 86, 94, 168, 197, 201, 285, 370, 371
**GONIOREFLECTOMETER**
COHERENT SOURCE
15, 71, 78, 84, 85, 131, 180, 191, 192, 194, 201, 206, 208, 283
INCOHERENT SOURCE
4, 35, 45, 47, 48, 58, 73, 83, 106, 139, 145, 163, 196, 197, 212, 218, 221, 235, 259, 295, 297, 300, 320, 323, 354, 371

R, Th	1. Adorjan, A. S. and Wierum, F. A., Radiative properties of rough surfaces, AIAA Paper No. 70–862, AIAA 5th Thermophysics Conference, June 1970.
Em, Th	2. Agababov, S. G., Effect of roughness of the surface of a solid body on its radiation properties and methods for their experimental determination, High Temp. **6** (1), 76–85 (1968).
SM	3. Agababov, S. G. and Eksler, L. I., Influence of the geometric characteristics of the relief of the surface of a solid on its radiation properties (determination of the roughness factor), High Temp. **9** (3), 475–479 (1971).
R, Ex	4. Agnew, J. T. and McQuistan, R. B., Experiments concerning infrared diffuse reflectance standards in the range 0.8 to 20.0 microns, J. Opt. Soc. Am. **43** (11), 999–1007 (1953).
S, R, Th	5. Aksenov, V. I., On the scattering of electromagnetic waves from sinusoidal and trochoidal surfaces of finite conductivity, Radio Eng. & Electron. **3** (4), 1–10 (1958).
S, Ex, Ap	6. Aksenov, V. I., Experimental investigation of electromagnetic wave scattering from periodically uneven surfaces, Radio Eng. & Electron. (USSR) **5** (5) 113–129 (1960).
S, Th	7. Aksenov, V. I., Utilization of Kirchhoff’s approximation in the problem of the scattering of radio waves by periodically rough surfaces with finite conductivity, Radio Eng. & Electron. **3**, 307–314 (Mar. 1961).
H	8. Amar, R. C. and Edwards, D. K., Reflection and transmission by rough-walled passages, AIAA Paper No. 72-303, 1–9, AIAA 7th Thermophysics Conference, San Antonio, Tex., Apr. 10–12, 1972.
R, Th	9. Ament, W. S., Toward a theory of reflection by a rough surface, Proc. IRE **41**, 142–146 (Jan. 1953).
R, S, Th	10. Ament, W. S., Forward- and back-scattering from certain rough surfaces, IRE Trans. **AP-4** (3), 369–373 (July 1956).
R, S, Th	11. Ament, W. S., Reciprocity and scattering by certain rough surfaces, IRE Trans. on Antennas and Propagation **AP-8** (2), 167–174 (Mar. 1960).
SM	12. American Standard, Surface texture-surface roughness, waviness and lay-ASA B46. 1–1962, UDC 621.9.015 -American Society of Mechanical Engineers.
SM	13. Armand, G., Lapujoulade, J., and Paigne, J., A theoretical relationship between the leakage of gases through the interface of two metals in contact and their superficial micro-geometry, Vacuum **14**, 53–57 (1964).
S, Th	14. Artmann, K., Zur Theorie der anomalen reflexion von optischen strichgittern, Z. Physik **119**, 529–567 (1942).
Ap, Ex(L)	15. Bair, M. E., Carmer, D. C, and Stewart, S. R., A gonioreflectometer facility using coherent and incoherent sources, Tech. Rept. AFAL–TR–70–161, p. 1–30. Infrared and Optics Lab., Univ. of Michigan, Ann Arbor, Mich. (Aug. 1970).
SM	16. Barash, V. Ya. and Uspenskii, Yu. P., Test metrological characteristics of surface-roughness measuring instruments, Measurement Tech. No. 2, 273–274 (Feb. 1969). (Translated from Izmeritel’naya Tekhnika, No. 2, 83, Feb. 1969).
S, Th	17. Baret, J. F. and Mann, J. A., Role of high-order grating effects in the scattered irradiance of corrugated surfaces, Appl. Opt. **10** (5), 1174–1176 (1971).
R, Th, Ex	18. Barkas, W. W., Analysis of light scattered from a surface of low gloss into its specular and diffuse components, Proc. Phys. Soc. (London) **51**, 274–295 (1939).
R, S, Th	19. Barrick, D. E., Rough surface scattering based on the specular point theory, IEEE Trans. Antennas and Propagation **AP-16**, 449–454 (1968).
R, Th	20. Barrick, D. E., Relationship between slope probability density function and the physical optics integral in rough surface scattering, Proc. IEEE **56**, No. 10, 1728–1729 (Oct. 1968).
R, S, Th	21. Barrick, D. E., Correction to “Rough surface scattering based on the specular point theory”, IEEE Trans. on Antennas and Propagation **AP-17** (1), 81 (1969).
S, Th	22. Barrick, D. E., Low-frequency scatter from a semielliptic groove in a ground plane, J. Opt. Soc. Am. **60** (5), 625–634 (May 1970).
S, Th	23. Barrick, D. E. and Peake, W. H., Scattering from surfaces with different roughness scales: analysis and interpretation, Rept. No. BAT–197A–10–3, Battelle Memorial Institute, Columbus, Ohio (1967). AD–662 751.
S, Th	24. Bass, F. G., On the theory of combination scattering of waves on a rough surface, AFCRL No. 511, 1–12, 1961. (Translated from Izvestiia VUZ, Radiofizika, **4** (1), 58–66, 1961). AD–262 417.
S, Th	25. Bass, F. G. and Bocharov, V. G., On the theory of scattering of electromagnetic waves from a statistically uneven surface, Radio Eng. & Electron. (USSR), **3** (2), 251–258 (1958).
Su(Th) R, T, Ex	26. Beaglehole, D. and Hunderi, O., Study of the interaction of light with rough metal surfaces. I. Experiment, Phys. Rev. B **2** (2), 309–321 (1970).
S, Ex	27. Beard, C. I., Coherent and incoherent scattering of microwaves from the ocean, IRE Trans. on Antennas and Propagation **AP-9** (5), 470–483 (Sept. 1961).
P, Th	28. Beckmann, P., The depolarisation of electromagnetic waves scattered from rough surfaces, Acta Technica No. 6, 511–523 (1961).
S, R, Th	29. Beckmann, P., The scattering of waves by a periodic surface, Czech. J. Phys. **B11**, 863–870 (1961).
R, S, Th	30. Beckmann, P., Shadowing of random rough surfaces, IEEE Trans. on Antennas and Propagation **AP–13**, 384–388 (May 1965).
S, R, Th	31. Beckmann, P., Scattering from rough surfaces for finite distances between transmitter and receiver, Rept. No. D1–82–0657, 1–39, Boeing Scientific Research Labs., Seattle, Wash., Geoastrophysics Lab. (Oct. 1967). AD–666 593.
R, Th, Su	32. Beckmann, P. and Spizzichino, A., The scattering of electromagnetic waves from rough surfaces, Vol. **4**, International Series of Monographs on Electromagnetic Waves (Pergamon Press, Macmillan, New York, N.Y. 1963).
R, Ex	33. Bennett, H. E., Specular reflectance of aluminized ground glass and the height distribution of surface irregularities, J. Opt. Soc. Am. **53** (12), 1389–1394 (1963).
R, Th	34. Bennett, H. E., Influence of surface roughness, surface damage, and oxide films on emittance, NASA SP–55, Symposium on Thermal Radiation of Solids (S. Katzoff, Ed.) pp. 145–152, NASA (1965).
Ex, R	35. Bennett, H. E., Measurement of Light scattered from very smooth surfaces, Preprint of a paper presented at the Optical Society of America 1970 Annual Meeting, Sept. 28–Oct. 2, held at Hollywood, Fla.
R, Th, Ex	36. Bennett, H. E. and Porteus, J. O., Relation between surface roughness and specular reflectance at normal incidence, J. Opt. Soc. Am. **51** (2), 123–129 (Feb. 1961).
R, Th	37. Berreman, D. W., Anomalous reststrahl structure from slight surface roughness, Phys. Rev. **163** (3), 855–864 (Nov. 1967).
R, Th	38. Berreman, D. W., Resonant reflectance anomalies: effect of shapes of surface irregularities, Preprint of 1969 spring meeting, Optical Society of America, San Diego, Calif., March 1969.
R, Th	39. Berreman, D. W., Reflectance and ellipsometry of slightly tarnished, dirty or rough surfaces, Bell Telephone Lab. Inc., Murray Hill, N.J., Preprint of paper ThG 17, Optical Society of America Annual Meeting, Chicago, Ill., Oct. 21–24, 1969.
R, Th	40. Barreman, D. W., Reflectance and ellipsometry when submicroscopic particles bestrew a surface, J. Opt. Soc. Am. **60** (4), 499–505 (Apr. 1970).
R, Th	41. Berry, E. M., Diffuse reflection of light from a matt surface, J. Opt. Soc. Am. **7**, 627–633 (1923).
H, Th	42. Bevans, J. T. and Edwards, D. K., Radiation exchange in an enclosure with directional wall properties, ASME Paper No. 64-WA/HT-52, 1–21, 1964 ASME Winter Annual Meeting, New York, N.Y.
R, Th	43. Biot, M. A., On the reflection of electromagnetic waves on a rough surface, J. Appl. Phys. **27** (9), 998 (Sept. 1956).
R, Th	44. Biot, M. A., Some new aspects of the reflection of electromagnetic waves on a rough surface, J. Appl. Phys. **28** (12), 1455–1463 (Dec. 1957).
R, Th, Ex	45. Birkebak, R. C., Monochromatic directional distribution of reflected thermal radiation from roughened surfaces, Ph. D. Dissertation, University of Minnesota, Minneapolis, Minn., 1962.
R, Ex	46. Birkebak, R. C., Dawson, J. P., McCullough, B. A., and Wood, B. E., Hemispherical reflectance of metal surfaces as a function of wavelength and surface roughness, Int. J. Heat Mass Transfer **10**, 1225–1232 (1967).
R, Th, Ex	47. Birkebak, R. C. and Eckert, E. R. G., Effects of roughness of metal surfaces on angular distribution of monochromatic reflected radiation, J. Heat Transf., Trans. ASME **C87**, 85–93 (1965).
R, Ex	48. Birkebak, R. C., Sparrow, E. M., Eckert, E. R. G., and Ramsey, J. W., Effect of surface roughness on the total hemispherical and specular reflectance of metallic surfaces, J. Heat. Transf., Trans. ASME **C86 (2)**, 193–199 (May 1964).
Em, Th, Ex	49. Black, W. Z. and Schoenhals, R. J., A study of directional radiation properties of specially prepared V-groove cavities, Trans. ASME (J. Heat Transf.) **90C** (4), 420–428 (Nov. 1968).
H, Ex	50. Black, W. Z. and Schoenhals, R. J., An experimental study of radiation heat transfer from parallel plates with direction-dependent properties, ASME Paper No. 70-HT/SpT-1, 1–6 (1970).
R, Th	51. Blakely, J. M. and Olson, D. L., Intensity distribution from a sinusoidal grating and application to diffusion measurements, Appl. Phys. **39** (7), 3476–3479 (June 1968). AD-682 663.
H, Th	52. Bobco, R. P., A script-F matrix formulation for enclosures with arbitrary surface emission and reflection characteristics, ASME Paper No. 70-HT/SpT-3, 1–8 (1970).
S, Th	53. Bocharov, V. G. and Bass, F. G., On the scattering of electromagnetic waves from a statistically inhomogeneous surface, Radio Eng. & Electron. (USSR), **3** (4), 184–187 (1958).
R, Th	54. Boerner, W. M., Compatibility studies. Vol. IV. appendix 3, Scattering of a beam with finite beam width from an impedance surface, Rept. No. 68-16, pp. 1–208, School of Electrical Engineering, Pennsylvania Univ. (Feb. 1968). AD-829 846.
R, Th	55. Bonzel, H. P. and Gjostein, N. A., Diffraction theory of sinusoidal gratings and application to in situ surface self-diffusion measurements, J. Appl. Phys. **39** (7), 3480–3491 (June 1968).
S, Th	56. Bowman, J. J., Senior, T. B. A., and Uslenghi, P. L. E., Electromagnetic and acoustic scattering by simple shapes, Wiley Interscience Div. (John Wiley and Sons, Inc., New York, N.Y., 1970). AD-699 859.
R, Th	57. Brand, K. W. and Spagnolo, F. A., Lambert diffuse reflection from general quadric surfaces, Rept. No. RE-278J, 1–30, Grumman Aircraft Engineering Corp., Bethpage, N.Y., Research Dept. (Jan. 1967). AD-805 421.
R, Ex	58. Brandenburg, W. M. and Neu, J. T., Undirectional reflectance of imperfectly diffuse surfaces, J. Opt. Soc. Am. **56** (1), 97–103 (Jan. 1966).
S, Th	59. Brekhovskikh, L. M., Diffraction of waves on a rough surface I. General theory, Zhurnal Eksperimental’nol I Theoreticheskoi Fiziki (J. Exptl. Theo. Phys. USSR) **23**, 275–288 (1952). Translated by Goss, R. N., U.S. Naval Electronics Lab., San Diego, Calif., in Three papers on diffraction of waves from a rough surface. pp. 1–22a.
S, Th	60. Brekhovskikh, L. M., Diffraction of waves on a rough surface II. Applications of the general theory, Zhurnal Eksperimental’nol I Theoreticheskoi (J. Exptl. Theo. Phys. USSR) **23**, 289–304 (1952). Translated by Goss, R. N., U.S. Naval Electronics Lab., San Diego, Calif., In Three papers on diffraction of waves from a rough surface. pp. 23–38g.
SM	61. Broadston, J. A., Education for surface texture assessment, Proceedings 1967–68 of International Conference on Properties and Metrology of Surfaces, Vol. **182**, Part 3K. Institution of Mechanical Engineers, London (Millson, R. J., Ed.).
R, Th	62. Brockelman, R. A. and Hagfors, T., Note on the effect of shadowing on the backscattering of waves from a random rough surface, IEEE Trans. on Antennas and Propagation, **AP-14**, 621–629 (Sept. 1966).
S, Ex	63. Bruehlman, R. J., Thomas, L. W., and Gonick, E., Effect of particle size and pigment volume concentration on hiding power of titanium oxide, (E. I. du Pont de Nemours and Co., Inc.), Official Digest, 252–267 (Feb. 1961).
SM	64. Bryan, J., Resume and critique of papers in part eight, International Research in Production Engineering, pp. 647–658 (1963). Published by ASME.
SM	65. Bryan, J. B. and Mohl, O., Microinterferometric scanning for full surface metrology, ASME Paper No. 64-WA/PROD-15, 1–7, Winter Annual Meeting, New York, N.Y., Nov. 29–Dec. 4, 1964 of ASME.
SM	66. Bufalini, J. J., An Investigation of methods of determining surface roughness of metals, Master Thesis, Dept. of Chemistry, Univ. of Arkansas (1958).
R, Th	67. Bullington, K., Reflection coefficients of irregular terrain, Proc. IRE **42**, 1258–1262 (Aug. 1954).
S, Th	68. Burke, J. E. and Twersky, V., On Scattering of waves by an elliptic cylinder and by a semielliptic protuberance on a ground plane, J. Opt. Soc. Am. **54** (6), 732–744 (June 1964).
SM	69. Butler, R. D. and Pope, R. J., Surface roughness and lubrication in sheet metal working, pp. 162–170, PMS* Proceedings, 1967–68.
Em	70. Caren, R. P. and Liu, C. K., Thermal radiation from a microscopically roughened dielectric surface, J. Heat Transfer, 73–79 (Feb. 1972).
Ap(L)	71. Carpenter, R. L. and Dalton, W. A. J., A laser bidirectional reflectometer, Rept. MDC 69-023, pp. 1–10, McDonnell Aircraft Company, McDonnell Douglas Corp. (1969).
R, Ex	72. Chaabane, G., Pieroway, C, and Francis, J., Reflectance measurements of dielectric coatings on a conductor substrate, AIAA Paper No. 71-448 AIAA 6th Thermophysics Conference, Tullahoma, Tenn., Apr. 26–28, 1971.
SM	73. Chamberlin, J. L., Centerline correction for precision roughness specimens, J. Res. Nat. Bur. Stand. (U.S.) **69C** (Eng. & Instr.), No. 4, 303–305 (Oct.–Dec. 1965).
R, P	74. Chen, Hsi-Shu, and Nagaraja, C. R., Polarization of light on reflection by some natural surfaces, Brit. J. Appl. Phys. (J. Phys. D.) 1, Ser. 2, 1191–1200 (1968).
S, P, Ex(L)	75. Cheo, P. K. and Renau, J., Wavelength dependence of total and depolarized back-scattered laser light from rough metallic surfaces, J. Opt. Soc. Am. **59** (7), 821–826 (1969).
S, Th	76. Chia, R. C., The theory of radar scatter from the ocean, Rept. No. CRES-TR-112-1, 1-125, Kansas Univ. Lawrence center for Research (Oct. 1968). AD-849 139.
R, Th	77. Christie, A. W., The luminous directional reflectance of snow, J. Opt. Soc. Am. 43, 621–622 (1953).
R(Ex)L, Th	78. Christie, F. A. and Devriendt, A. B., Bidirectional reflectance from surfaces formed by the ruling of orthogonal parallel V-grooves, AIAA Paper No. 72-55, 1–9, AIAA 10th Aerospace Sciences Meeting, San Diego, Calif., Jan. 17–19, 1972.
S, Th	79. Chu, C. M. and Churchill, S. W., Multiple scattering by randomly distributed obstacles–Methods of solution, IRE Trans. **AP-4** (2), 142–148 (Apr. 1956).
SM	80. Chunin, B. A., Measuring depth of point defects in polished surfaces by means a linnik microinterferometer, Measurement Tech. No. 2, 276–277 (Feb. 1969). Translated from Izmeritel’naya Tekhnika, No. 2, 84–85 (Feb. 1969).
R, Th	81. Clarke, R. H., Theoretical characteristics of radiation reflected obliquely from a rough conducting surface, Proc. IEE **110** (1), 91–100 (Jan. 1963).
R, Th, Ex	82. Clarke, R. H. and Hendry, G. O., Prediction and measurement of the coherent and incoherent power reflected from a rough surface, IEEE Trans. on Antennas and Propagation, **AP-12** (3), 353–363 (1964).
R, Ap	83. Comstock, D. F., Reflectance measurement by three-dimensional goniometry, Preprint (Appl. Opt.) (Arthur D. Little, Inc., 1968).
R, Th, Ex(L)	84. Condie, M. A., An experimental investigation of the statistics of diffusely reflected coherent light, Tech. Report No. AFAL-TR-66-387, Oct. 1966.
R, Th, Ex	85. Considine, P. S., Cronin, D. J., and Reynolds, G. O., Angular dependence of radiance of rough surfaces in imaging systems, J. Opt. Soc. Am. 56 (7), 877–883 (July 1966).
S, P, Ex	86. Cooke, D. D. and Kerker, M., Light scattering from long thin glass cylinders at oblique incidence, J. Opt. Soc. Am. 59 (1), 43–48 (1969).
S(L), Th	87. Crane, R. B., Use of a laser-produced speckle pattern to determine surface roughness, J. Opt. Soc. Am. 60 (12), 1658–1663 (Dec. 1970).
R, Th	88. Crowell, J. and Ritchie, R. H., Surface-plasmon effect in the reflectance of a metal, J. Opt. Soc. Am. 60 (6), 794–799 (June 1970).
S, Th(L)	89. Dainty, J. C, Some statistical properties of random speckle patterns in coherent and partially coherent illumination, Optica Acta 17 (10), 761–772 (1970).
R, Th	90. Daniels, F. B., Radar determination of the scattering properties of the moon, Nature **187** No. 4735, 399 (July 1960).
R, Th	91. Davies, H., The reflection of electromagnetic waves from a rough surface, Proc. Inst. Elec. Engr. **101**, 118 (1954).
R, Th	92. Davies, H., The reflection of electromagnetic waves from a rough surface, Proc. IEE **101**, 209–214 (1954).
R, Ex	93. Davies, J. M. and Zagieboylo, W., An integrating sphere system for measuring average reflectance and transmittance, Appl. Opt. 4 (2), 167–174 (1965).
R, Ex	94. Drummeter, L. F., Jr., and Fowler, W. B., Some angular reflectance properties of light-trapping surfaces, NRL Report No. 5753, 1–14, U.S. Naval Research Lab. (1962). AD-276 052.
SM	95. Dunin-Barkovsky, I. V., Analysis of surface irregularities by the spectral method, pp. 211–215, PMS* Proceedings, 1967–68.
R, Su	96. Dunn, S. T., A possible approach to evaluation of flux scattered by roughened surfaces, AIAA 3rd Aerospace Sciences Meeting, AIAA Paper No. 66–20 (Jan. 1966).
R, Em Su(Ex)	97. Dunn, S. T., Richmond, J. C., and Parmer, J. F., Survey of infrared measurement techniques and computational methods in radiant heat transfer, J. Spacecraft and Rockets, 962–975 (July 1966).
SM	98. Dupuy, M. O., High-precision optical prorilometer for the study of micro-geometrical surface defects, pp. 255–259, PMS* Proceedings, 1967–68.
SM	99. Eberle, O. A., Graphical method for statistical assessment of surface roughness comparison specimens, pp. 368–396, PMS* Proceedings, 1967–68.
R, Su	100. Edward, D. K., Radiative transfer characteristics of materials, J. Heat Transfer, Trans. ASME **C91**, 1–15 (1969).
H, Th	101. Edwards, D. K. and Bertak, I. V., Imperfect reflections in thermal radiation transfer, AIAA Paper No. 70-860, AIAA 5th Thermophysics Conference, June 1970.
R, Em, Th, Ex	102. Edwards, D. K. and Catton, I., Radiation characteristics of rough and oxidized metals, Symposium on Thermophysical properties, held at Purdue Univ., 1965, pp. 189–199.
P(L), Ex	103. Egan, W. G. and Hallock, H. B., Coherence-polarization, 1–38, Grumman Aircraft Engineering Corp., Bethpage, N.Y., Research Dept. (Mar. 1969). AD-849 398.
S, R, Th	104. Erma, V. A., Exact solution for the scattering of electromagnetic waves from bodies of arbitrary shape. III. obstacles with arbitrary electromagnetic properties, Phys. Rev. **179** (5), 1238–1246 (1969).
S, Ex(L)Th	105. Estes, L. E., Narducci, L. M., and Tuft, R. A., Scattering of light from a rotating ground glass, J. Opt. Soc. Am. **61** (10), 1301–1306 (Oct. 1971).
R, Ex, Ap	106. Euler, J., Reflexionsmessungen an gesandetem aluminium, Z. Angen Physik **1** (12), 569–573 (1949).
R, Th	107. Evans, L. B., Chu, C. M., and Churchill, S. W., The effect of anisotropic scattering on radiant transport J. Heat Transfer Trans. ASME **C87** (3), 381–387 (1965).
S, R, Th	108. Fante, R. L., Discussion of a model for rough surface scattering, IEEE Trans, on Antennas and Propagation, **AP-13** (4), 652–653 (1965).
R, Th	109. Fedders, P. A., Surface roughness and the absorption of electromagnetic waves in simple metals, Phys. Rev. **181** (3), 1053–1058 (1969).
S, Th	110. Felsen, L. B., Comment on the paper, The scattering of electromagnetic waves by a corrugated sheet, by T. B. A. Senior, Can. J. Phys. **37**, 1563–1565 (1959).
SM	111. Felstead, E. B., Additional optical data processing techniques for surface roughness studies, Proc. IEEE **57** (3), 343–344 (Mar. 1969).
R, Th	112. Fenstermaker, C. A. and McCrackin, F. L., Errors arising from surface roughness in ellipsometric measurement of the refractive index of a surface, Surface Sci. **16**, 85–96 (1969).
H	113. Fischer, W. D. and Hering, R. G., Radiant heat transfer between nongray directional surfaces, AIAA Paper No. 72-306, 1–7, AIAA 7th Thermophysics Conference, San Antonio, Tex., Apr. 10–12, 1972.
R, S, Su	114. Friedhoffer, J. A., Scattering of electromagnetic waves by rough surfaces, Rept. No. BRL-MR-1805, 1–29, Ballistic Research Labs., Aberdeen Proving Ground, Md. (Nov. 1966). AD-813 377.
SM	115. Funai, A. I. and Rolling, R. E., Inspection techniques for characterization of smooth, rough, and oxidized surfaces, AIAA Progress in Aeronautics and Astronautics: Thermophysics of Spacecraft and Planetary Bodies (G. B. Heller, ed.), **20**, 41–64 (Academic Press, New York, N.Y., 1967).
R, S, Th	116. Fung, A. K., Theory of radar scatter from rough surfaces, bistatic and monostatic, with application to lunar radar return, J. Geophys. Res. **69** (6), 1063–1073 (Mar. 1964).
S, P, Th	117. Fung, A. K., Scattering and depolarization of EM waves from a rough surface, Proc. IEEE **54**, 395–396 (1966).
P, Th	118. Fung, A. K., Character of wave depolarization by a perfectly conducting rough surface and its application to earth and moon experiments, Planet. Space Sci. **15**, 1337–1347 (1967), Pergamon Press Ltd., Printed in Northern Ireland.
R, S, P, Th	119. Fung, A. K. and Chan, H. L., Backscattering of waves by composite rough surfaces, IEEE Trans, on Antennas and Propagation **AP-17** (5), 590–597 (1969).
S, Th	120. Fung, A. K. and Chan, H. L., On the integral for backscattering from a randomly rough surface, Proc. IEEE **59** (8), 1280–1281 (Aug. 1971).
S Th	121. Fung, A. K. and Moore, R. K., The correlation function in Kirchoff’s method of solution of scattering of waves from statistically rough surfaces, J. Geophys. Res. **71** (12), 2939–2943, June 15, 1966.
S, P, Th	122. Fung, A. K., Moore, R. K., and Parkins, B. E., Notes on backscattering and depolarization by gently undulating surfaces, J. Geophys. Res. **70** (6), 1559–1562, Mar. 15, 1965.
S, Th(L)	123. Gabor, D., Laser speckle and its elimination, IBM J. Res. & Develop. **14** (5), 509–514 (1970).
S, P, Th	124. George, J. L., Back-scattering of circularly polarized waves from slightly rough surfaces, Rept. No. 1179-1, Antenna Lab. Ohio State Univ. Research Foundation, pp. 1–11 (Apr. 1961). AD-407 424.
Su	125. Ginsberg, I. W., Limperis, T., Nicodemus, F. E., and Richmond, J. C., Geometrical considerations in reflectance nomenclature, to be published as Nicodemus, F. E., Richmond, J. C., Hsia, J. J., Venable, W. H., Jr., Ginsberg, I. W. and Limperis, T. Geometrical considerations and nomenclature for reflectance. NBS Monograph.
S, Th.	126. Goldfischer, L. I., Autocorrelation function and power spectral density of laser-produced speckle patterns, J. Opt. Soc. Am. **55** (3), 247–253 (Mar. 1965).
SM	127. Gorodinskii, G. M., Device for the production inspection of the surface roughness of plate glass, Measurement Techniques No. 4, 413–415 (July–Aug. 1958).
R, Th, Ex	128. Gorodinskii, G. M., The statistical interference of light upon reflection from matte-glass surfaces, Opt. Spectrosc. **15**, 57–60 (1963).
P, Ex	129. Gorodinskii, G. M., Polarizing properties of frosted glass surfaces in the case of reflection, Opt. Spectrosc. **16**, 59–60 (1964).
R, Ex	130. Gorton, A. F., Reflection from, and transmission through, rough surfaces, Phys. Rev. **7** (1), 66–78 (1916).
S, Ex(L)	131. Gould, G., et al., Coherent detection of light scattered from a diffusely reflecting surface, Appl. Opt. **3**, 648–649 (1964).
S	132. Gravatt, C. C., Jr., The applications of light scattering Appl. Spectroscopy **25** (5), 509–517 (1971).
SM	133. Green, E., Review of surface texture measurement and the associated metrological problems, pp. 330–343, PMS* Proceedings, 1967–68.
S, Th, Ex(L)	134. Greenwood, D. P. and Powers, E. J., Determination of surface-height power spectral density from a laser-scattering measurement, J. Opt. Soc. Am. **61** (11), 1589 (Nov. 1971).
SM	135. Greenwood, J. A. and Williamson, J. B. P., Contact of nominally flat surfaces, Proc. Roy. Soc. **A295**, 300–319 (1965).
SM	136. Guentert, O. J., Development of surface measurement system, Raytheon Co. Waltham, Mass., Research Div. Rept. No. S-815, 1–40 (Sept. 1965). AD-472 415.
R, S, Th	137. Hagfors, T., Relationship of geometric optics and and auto-corelation approaches to the analysis of lunar and planetary radar echoes, J. Geophys. Res. **71**, 379–383 (Jan. 1966).
S, Th	138. Hansen, J. E. and Cheyney, H., Theoretical spectral scattering of clouds in the near infrared, J. Geophys. Res. **74** (13), 3337–3346 (1969).
R, Th	138a. Hapke, B. W., A theoretical photometric function for the lunar surface, J. Geophys. Res. **68** (15), 4571–4586 (1963).
R, S, Ex	139. Harten, H., Zur Streureflexion an matten Oberflächen, Z. Physik **126**, 27–34 (1949).
S, Th	140. Hayre, H. S. and Moore, R. K., Theoretical scattering coefficient for near vertical incidence from contour maps, J. Res. Nat. Bur. Stand. (U.S.), **65D** (Radio Prop.) No. 5, 427–432 (Sept.–Oct. 1961).
H	141. Hering, R. G. and Fischer, W. D., Radiant heat transfer between nongray surfaces, AIAA Paper No. 71-464 AIAA 6th Thermophysics Conference, Tullahoma, Tenn., Apr. 26–28, 1971.
R, Th	142. Hering, R. G. and Smith, T. F., Apparent radiation properties of a rough surface, AIAA Paper No. 69-622, AIAA 4th Thermophysics Conference, June 1969.
H, Th	143. Hering, R. G. and Smith, T. F., Surface roughness effects on equilibrium temperature of interacting surfaces, AIAA Paper No. 70-817, AIAA 5th Thermophysics Conference, June 1970.
H, Th	144. Hering, R. G. and Smith, T. F., Surface roughness effects on radiant energy interchange, ASME Paper No. 70-HT/SpT-2, 1–9 (1970).
R, Th, Ex	145. Herold, L. M. and Edwards, D. K., Bidirectional reflectance characteristics of rough, sintered-metal, and wire-screen surface systems, AIAA Paper No. 66-19, AIAA 3rd Aerospace Sciences Meeting, Jan. 1966.
S, Th, Ex	146. Hiatt, R. E., Senior, T. B. A. and Weston, V. H., A study of surface roughness and its effect on the backscattering cross section of spheres, Proc. IRE **48** (12), 2008–2016 (Dec. 1960).
S, Th	147. Hills, N. L. and Karp, S. N., Semi-infinite diffraction gratings. I., Commun. Pure Appl. Math. **18** (1/2), 203–233 (Feb.–May 1965). AD-617 220.
Em, Ex	148. Hires, R. G. and Evans, R. C., Study of surfaces designed to increase thermal radiation, Report No. TG-618, Applied Physics Laboratory, Johns Hopkins University, Silver Spring, Md. (Nov. 1964). AD-459 468.
SM	149. Hodgkinson, I. J., A simple scatter method for optical surface roughness and slope measurements. Roughness of polished fused silica, J. Phys. E (Sci. Instrum.) **3** (5), 341–342 (May 1970).
S, Th	150. Hoffman, W. C., Scattering of electromagnetic waves from rough surfaces, Quart. Appl. Math. **13**, 291–304 (Oct. 1955).
S, Ex	151. Hottel, H. C., Sarofim, A. F., Dalzell, W. H. and Vasalos, I. A., Optical properties of coatings: effect of pigment concentration, AIAA Journal, 1395–1398 (Oct. 1971).
S, Ex, Th	152. Hottel, H. C., Sarofim, A. F., Vasalos, I. A. and Dalzell, W. H., Multiple scatter: comparison of theory with experiment, ASME Paper No. 69-WA/HT-44, 1–7, ASME Winter Annual Meeting, Nov. 16–20, 1969.
R, Th	153. Houchens, A. and Hering, R. G., Bidirectional reflectance of rough metal surfaces, AIAA Progress in Aeronautics and Astronautics: Thermophysics of Spacecraft and Planetary Bodies (G. B. Heller, ed.) **20**, 65–89 (Academic Press, New York, N.Y., 1967).
H	154. Houchens, A. F., Hering, R. G. and Savage, L. D., Roughness effects on radiant heat transfer between interacting surfaces, AIAA Paper No. 72-305, AIAA 7th Thermophysics Conference, San Antonio, Tex., Apr. 10–12, 1972.
H	155. Howell, J. R. and Durkee, R. E., Comparison of analysis and experiment for radiative transfer between surfaces with collimated incident radiation, ASME Paper No. 70-HT/SpT-23, Am Soc. Mech. Engr. (1970).
R, Em, Th	156. Howell, J. R. and Perlmutter, M., Directional behavior of emitted and reflected energy from a specular, gray, asymmetric groove, NASA TND-1874 (1963).
SM	157. Hsia, J. J. G., Electrolytic preparation of reproducible metallic surfaces, Master thesis, Dept. of Mechanical Engineering, Purdue Univ., 1964.
Ma	158. Hufford, G., An integral equation approach to the problem of wave propagation over an irregular surface, Quart. Appl. Math. **9**, (4), 391–403 (1952).
R(Th)	159. Hunderi, O. and Beaglehole, D., Study of the interaction of light with rough metal surfaces. II. Theory, Phys. Rev. B **2** (2), 321–329 (1970).
S, Th	160. Isakovich, M. A., Scattering of waves from a statistically rough surface, Zhurnal Eksperimental’noi I Theoreticheskoi Fiziki (J. Exptl. Theo. Phys. USSR) **23**, 305–314 (1952). Translated by Goss, R. N., U.S. Naval Electronics Lab., San Diego, Calif., in Three papers on diffraction of waves from a rough surface. pp. 39–52c.
R, Th	161. Itoh, T. and Mittra, R., An Analytical study of the echelette grating with application to open resonators, IEEE Tran. on Microwave Theory and Techniques **VMTT-17** (6), 319–327 (June 1969). AD-696 236.
SM	162. Janssen, J. E. and Schmidt, R. N., The measurement of total surface area, NASA SP-55, Symposium on Thermal Radiation of Solids (S. Katzoff, ed.), pp. 179–181, NASA (1965).
S, Ex, Ap	163. Johnson, M. C., Vacuum ultraviolet scattering distributions, Appl. Opt. **7** (5), 879–881 (1968).
S, Th	164. Jones, A. R., Scattering of focused electromagnetic waves by infinite cylinders, J. Phys. A (Gen. Phys.) Great Britain Ser. 2, **2**, 311–322, 1969.
S, Th, Ex	165. Judd, D. B., Optical specification of light scattering materials, J. Res. Nat. Bur. Stand. (U.S.) **19**, 287–317 (Sept. 1937). RP1026.
Ma	166. Kac, M., On the average number of real roots of a random algebraic equation, Bull. Math. Soc. **49**, 314–320 (1943).
R, Th	167. Kalhor, H. A. and Neureuther, A. R., Numerical method for the analysis of diffraction gratings, J. Opt. Soc. Am. **61** (1), 43–48 (1971).
Em, Th, Ex, Ap	168. Kanayama, K., Apparent directional emittances of V- groove and circular -groove rough surfaces, Heat Transfer-Japanese Res. **1** (1), 11–22 (Jan.–Mar. 1972).
S(L), Ex	169. Karinskii, S. S., Komarov, V. G. and Mondikov, V. D., Scattering of light by elastic surface waves, JETP Letters **9** (7), 225–226, 1969. (Original ZhETF Pis. Red. **9** (7), 380–383, 1969.)
R, Th	170. Kaufman, D. E., A vector solution for electromagnetic wave scattering from a rough surface of arbitrary dielectric constant, Radio Sci. **6** (1), 7–20 (1971).
R, Ex, SM	171. Keegan, H. J., Schleter, J. C., Weidner, V. R., Spangenberg, D. B., Strang, A. G. and Chamberlin, J. L., Effect of surface texture on diffuse spectral reflectance, NASA SP-55, Symposium on Thermal Radiation of Solids (S. Katzoff, ed.) pp. 165–177, NASA (1965).
R, Ex	172. Kelly, F. J. and Moore, D. G., A test of analytical expressions for the thermal emissivity of shallow cylindrical cavities, Appl. Opt. **4** (1), 31–40 (1965).
S, Su	173. Kerker, M., The scattering of light and other electromagnetic radiation (Academic Press, New York, 1969).
S, Th	174. Kerker, M., Cooke, D. D. and Carlin, J. M., Light scattering from infinite cylinders, the dielectric-needle limit, J. Opt. Soc. Am. **60** (9), 1236–1239 (Sept. 1970).
R, Th	175. Kholopov, G. K., Calculation of emissivities of non-diffuse cavity-type sources, Soviet J. Opt. Technology **35** (1), 1–6 (1968).
SM	176. Kimoto, S. and Russ, J. C., The characteristic and applications of the scanning electron microscope, Am Scientists **57** (1), 112–133 (1969).
S, Th	177. King, R. W. P. and Harrison, C. W. Jr., Scattering by imperfectly conducting spheres, IEEE Trans. on Antennas and Propagation **AP-19** (2), 197–207 (1971).
R, Th, Su	178. Kirtikos, H., Lang, K. C. and Boerner, W., et al, Investigation of rough earth propagation models, RADC-TR-67-531, 10–18 (Oct. 1967). AD-822 904.
R, S, Th	179. Kivelson, M. and Moszkowski, S., Reflection of electromagnetic waves from a rough surface, J. Appl. Phys. **36**, 3609–3612 (1966).
Ap	180. Kneissl, G. J. and Richmond, J. C., A laser-source integrating sphere reflectometer, Nat. Bur. Stand. (U.S.), Tech. Note 439, 54–63 (Feb. 1968).
S, Th	181. Kodis, R. D., A note on the theory of scattering from an irregular surface, IEEE Trans, on Antennas and Propagation, **AP-14**, 77–82 (Jan. 1966).
S, Th, Ex	182. Kozlov, V. P. and Federova, E. O., Reflection of light from a scattering medium, Soviet J. Opt. Technology **34** (1), 1–6 (1967).
S, Th	183. Kroll, N. M. and Kelly, P. L., Temporal and spatial gain in stimulated light scattering, Phys. Rev. A **4** (2), 763–776 (1971).
S, Th	184. Kubelka, P., New contributions to the optics of intensely light-scattering materials. Part I, J. Opt. Soc. Am. **38** (5), 448–457 (May 1948).
T, S, Th	185. Kurtz, C. N., Transmittance characteristics of surface diffusers and the design of nearly band-limited binary diffusers, J. Opt. Soc. Am. **62** (8), 982–989 (Aug. 1972).
R, Ex	186. Lagrone, A. H. and Tolbert, C. W., Reflection studies of millimeter and centimeter radio waves for Gulf of Mexico paths, Rept. No. EERL-64, 1–38, Texas Univ. Austin Electrical Engineering Research Lab. (Oct. 1952). AD-494 931.
S, Th	187. Lam, J., Scattering by an expanding conducting sphere, J. Math. Phys. (Cambridge) **47** (2), 199–212 (1968).
S, Th	188. Lang, K. C., Diffraction of electromagnetic waves by a moving impedance wedge, Radio Sci. **6** (6), 655–663 (1971).
S(L)	189. Langmuir, R. V., Scattering of laser light, Appl. Phys. Letters **2** (2), 29–30 (January 15, 1963).
SM	190. Latanision, R. M., Characterization of metal surfaces, RIAS Tech. Rept. 71–08c, Research Institute for Advanced Studies, Martin Marietta Co., Baltimore, Md. (1971). AD-722 013.
R, Ex, Th	191. Latta, M. R., The scattering of 10.6 micron radiation from ground glass surfaces, Preprint of 1969 spring meeting. Optical Society of America, San Diego, Calif., March 1969.
R, Th Ex(L)	192. Leader, J. C, Bidirectional scattering of electromagnetic waves from rough surfaces, MDC 70-022, McDonnell Aircraft Co., St. Louis, Mo. (1970).
S, Th	193. Leader, J. C., Multiple scattering of electromagnetic waves from rough surfaces, MDC 70-037, McDonnell Aircraft Co., St. Louis, Mo. (1970).
S, Th, Ex(L)	194. Leader, J. C., Hemispherical scattering of electromagnetic waves from rough surfaces, McDonnell Aircraft Co. Rept. No. MCAIR 72-037 (1972).
L	195. Libenson, M. N., Romanov, G. S., and Imas Ya. A., Temperature dependence of the optical constants of a metal in heating by laser radiation, Soviet Physics-Technical Physics 13 (7), 925–928 (Jan. 1969). Translated from Zhurnal Tekhnicheskoi Fiziki **38** (7), 1116–1119 (July 1968).
Ap	196. Lindberg, R. W., A specular gonioreflectometer, Proc. 21st Inst. Soc. Am. Conf., N.Y. 1966, **21** pt. 1, Paper No. 12.6-3-66.
R, Ex	197. Loehrlein, J. E., Winter, E. R. F., and Viskanta, R., Measurement of bidirectional reflectance using a photographic technique, AIAA Paper No. 70-859, AIAA 5th Thermophysics Conference, June 1970.
R, Th	198. Longuet-Higgins, M., Reflection and refraction of a random moving surface—II: Number of specular points on a Gaussian surface, J. Opt. Soc. Am. **50**, 845–850 (1960).
R, Th	199. Look, D. C. Jr. and Love, T. J., Investigation of the effects of surface roughness upon reflectance, AIAA Paper No. 70-820, AIAA 5th Thermophysics Conference, June 1970.
Em, Th	200. Love, T. J. and Turner, W. D., Directional emittance from emitting, absorbing, and scattering media, AIAA Paper No. 69-625, AIAA 4th Thermophysics Conference, June 1969.
S, Ex	201. Lundberg, J. L., Light scattering from large fibers at normal incidence, J. Colloid and Interface Sci. **29** (3), 565–583, 1969.
S, Th	202. Lynch, P. J., Curvature corrections to rough-surface scattering at high frequencies, J. Acoust. Soc. Am. **47** (3) part 2, 804–815 (1970).
S, Th	203. Lynch, P. J. and Wagner, R. J., Energy conservation for rough-surface scattering, J. Acoust. Soc. Am. **47** (3) part 2, 816–821 (1970).
R, Th	204. Lynch, P. J. and Wagner, R. J., Rough-surface Scattering: Shadowing, multiple scatter, and energy conservation, J. Math. Phys. **11** (10), 3032–3042 (1970).
R, T, Th	205. Marathay, A. S., Heiko, L. and Zuckerman, J. L., Study of rough surfaces by light scattering, Appl. Opt. **9** (11), 2470–2476 (1970).
R, Ex(L)	206. Massey, G. A., Photomixing with diffusely reflected light, Appl. Opt. **4** (7), 781–784 (July 1965).
S, Th	207. Mautz, J. R. and Harrington, R. F., Radiation and scattering from bodies of revolution, Appl. Sci. Res. **20**, 405–435 (June 1969).
R, Ex, Ap(L)	208. Maxwell, J. R., Target signature analysis center: DATA compilation eleventh supplement. Vol. 1: Bidirectional reflectance: Definition, discussion, and utilization., Report No. AFAL-TR-72-226, Target signature analysis center, infrared and optics Division, Willow Run Laboratories, The University of Michigan, Ann Arbor, Mich. (Oct. 1972).
S, Th	209. McClellan, R. P. and Stroke, G. W., Solution of the non-homogeneous Helmholtz equation for optical gratings with perfectly conducting boundaries, J. Math. Phys. **45**, 383–390 (1967).
P, Th	210. McCoyd, G. C., Polarization properties of a simple dielectric rough-surface model, J. Opt. Soc. Am. **57** (11), 1345–1350 (1967).
S, Th	211. Mclvor, I. K., Two-dimensional scattering of a plane compressional wave by surface imperfections, Bull. Seismol. Soc. Am. **59** (3), 1349–1364 (June 1969). AD-692 601.
R, Ex	212. McNicholas, H. J., Absolute methods in reflectometry, J. Res. Nat. Bur. Stand. (U.S.), **1**, 29–73 (1928). RP3.
R, Th	213. Meecham, W. C, A method for the calculation of the distribution of energy reflected from a periodic surface, IRE Trans. on Antennas and Propagation **AP-4** (3), 581 (July 1956).
R, Th	214. Meecham, W. C., Variational method for the calculation of the distribution of energy reflected from a periodic surface. I., J. Appl. Phys. **27** (4), 361–367 (1956).
R, Th	215. Meecham, W. C., Fourier transform method for the treatment of the problem of the reflection of radiation from irregular surfaces, J. Acoust. Soc. Am. **28** (3) 370–377 (1956).
S, R, Th	216. Merriam, R. L. and Viskanta, R., Radiative characteristics of absorbing, emitting, and scattering media on opaque substrata, J. Spacecraft **5** (10), 1210–1215 (1968).
R, Th, Ex	217. Middleton, W. E. Knowles and Mungal, A. G., The luminous directional reflectances of snow, J. Opt. Soc. Am. **42** (8), 572–579 (1952).
R, Ex	218. Middleton, W. E. Knowles and Wyszecki, G., Colors produced by reflection at grazing incidence from rough surfaces, J. Opt. Soc. Am. **47** (11), 1020–1023 (1957).
S, R, Th	219. Miles, J. W., On nonspecular reflection at a rough surface, J. Acous. Soc. Am. **26**, 191–199 (Mar. 1954).
S, Th	220. Millar, R. F., Rayleigh Hypothesis in scattering problems, Electronics Letters **5** (17), 416–418, 1969.
R, A_P_	221. Miller, E. R. and Vun Kannon, R. S., Development and use of a bidirectional spectroreflectometer, AIAA Paper No. 67-313, AIAA Thermophysics Specialist Conference, New Orleans, La. (Apr. 17–20, 1967).
S, Th	222. Mittra, R. and Wilton, D. R., Anumerical approach to the determination of electromagnetic scattering characteristics of perfect conductors, Proc. IEEE **57** (11), 2064–2065 (Nov. 1969).
R, Th	223. Mitzner, K. M., Theory of the scattering of electromagnetic waves by irregular interfaces, Rept. No. TR 30, pp. 1–131, Antenna Lab. Calif. Inst. Tech. (Jan. 1964). AD-436 811.
P, Th	224. Mitzner, K. M., Change in polarization on reflection from a tilted plane, Radio Science **1** (1), 27–29 (Jan. 1966).
R, P, Th	225. Monroe, R. L., Approximate step-function response of a horizontally polarized electromagnetic wave reflected at an imperfectly conducting surface, J. Appl. Phys. **41** (12), 4820–4822 (1970).
SM	226. Moody, J. C., Measurement of ultrafine surface finishes, Rept. No. SC-DC-67-1425, pp. 1–17, Sandia Corp. Albuquerque, N. Mex. (Mar. 1967).
SM	227. Motycka, J., Proposed interferometric method of measurement of roughness and autocorrelation function in smooth-finished surfaces, Appl. Opt. **8** (7), 1435–1438 (1969).
SM	228. Munnerlyn, C. R. and Latta, M., Rough surface interferometry using a CO_2_ laser source, Appl. Opt. **7** (9), 1858–1859 (1968).
SM	229. Munnerlyn, C. R., Givens, N. P., Hopkins, R. E., Interferometric measurements of optically rough surfaces, preprint of 1969 IEEE Conference on Laser Engineering and Applications, Washington, D.C., May 1969.
R, Th	230. Nagata, K., Yoshida, N., and Nishiwaki, J., Ensemble-averaged coherence function of light reflected from rough surface: determination of its correlation length, Japanese J. Appl. Phys. **9** (5), 505–515 (1970).
SM	231. Nara, J., Profile analyzer, Bull. Japan Soc. Prec. Engg. **3** (3), 59–61 (1969). (Also in Bull. Natl. Res. Lab. Metrology, Japan, No. 19, 18–20, Oct. 1969).
SM	232. Nara, J., Two-dimensional representation of surface roughness, Bull. Natl. Res. Lab. Metrology (Japan) No. 19, pp. 7–17 (Oct. 1969). (C.I.R.P. Vol. **XVII**, 485–495, Pergamon Press Ltd. 1969).
R, Th	233. Nelson, H. F., Ray reflection from rough dielectric surfaces, AA & ES 66–5, Purdue University, 1-142 (1966).
R, Th	234. Nelson, H. F. and Goulard, R., Reflection from a dielectric surface, J. Opt. Soc. Am. **57** (6), 769–771 (1967).
R, Ex	235. Neu, J. T. and Dummer, R. S., Theoretical and practical implications of the bidirectional reflectance of spacecraft surfaces, Paper No. 68-26, AIAA 6th Aerospace Sciences Meeting New York, N.Y. Jan. 22–24, 1968.
Em, Th	236. Nevskii, A. S. and Il’chukova, L. V., Effect of macroscopic roughness on the character of the emission from a surface, High Temp. **6** (6), 990–994 (1968).
R, Th	237. Nicodemus, F. E., Directional reflectance and emissivity of an opaque surface, Appl. Opt. **4** (7), 767–773, 818 (1965).
R	238. Nicodemus, F. E., Reflectance nomenclature and directional reflectance and emissivity, Appl. Opt. **9** (6), 1474–1475 (June 1970).
S, Th	239. Olaofe, G. O., Scattering by an arbitrary configuration of parallel circular cylinders, J. Opt. Soc. Am. **60** (9), 1233–1236 (Sept. 1970).
S(L)	240. Oliver, B. M., Sparkling spots and random diffraction, Proc. IEEE **51**, 220–221 (1963).
R, Th	241. Ornstein, L. S., and Van der Burg, A., Reflectivity of corrugated surfaces, Physica **IV** (11), 1181–1189 (1937).
S, Th	242. Parkins, B. E., Scattering from time-varying rough surfaces, Rept. No. TR-3, 1-18, Bell Telephone Labs., Inc., Whippany, N.J. (Sept. 1966). AD-656 549.
R, Em, Th	243. Parmer, J. F., and Wiebelt, J. A., The thermal radiation characteristics of specular walled grooves in the solar space environment, AIAA 4th Aerospace Sciences Meeting, Los Angeles, Calif., AIAA Paper No. 66-459 (June 1966).
S, Th	244. Peake, W. H., Barrick, D. E., Fung, A. K., and Chan, H. L., Comments on Backscattering of waves by composite rough surfaces, IEEE Trans. on Antennas and Propagation **AP-18** (5), 716–726 (Sept. 1970).
SM	245. Peklenik, J., New developments in surface characterization and measurements by means of random process analysis pp. 108–126, PMS* Proceedings 1967–68.
S, Th	246. Pimenov, Yu. V., and Kravtsova, G. V., Diffraction of a plane electromagnetic wave by an ideally conducting spherical segment, Radio Eng. **24** (2), 101–106, 1969.
SM	247. Piper, B., and Callis, W. P., Surface-finish measurement on non-ferrous materials, American Machinist Special Rept. No. 476, 105–120 (1959).
S, Th	248. Plonus, M. A., Scattering from small obstacles on an infinite conducting plane, Rept. 7741-3-T, 1-60, University of Michigan, Ann Arbor, Mich. (Dec. 1966). AD-814 843.
R, Th	249. Polgar, L. G., and Howell, J. R., Directional radiative characteristics of conical cavities and their relation to lunar phenomena, AIAA Progress in Aeronautics and Astronautics: Thermophysics and Temperature Control of Spacecraft and Entry Vehicles (G. B. Heller, ed.), (Vol. **18**, pp. 311-323, Academic Press, New York, N.Y., 1966).
S, Th	250. Pollon, G. E., Statistical parameters for scattering from randomly oriented arrays, cylinders, and plates, IEEE Trans. on Antennas and Propagation **AP-18** (1), 68–75 (Jan. 1970).
R, P, Ex	251. Polyanskii, V. K., Kovalskii, L. V., and Milovidova, T. I., Specular reflection from a mat surface with coarse microtopography, Opt. Spectrosc. **25** (5), 415–416 (1968).
P, Ex, Ap	252. Polyanskii, V. K., and Rvachev, V. P., On the reflection of light from rough surfaces, Opt. Spectrosc. **20** (4), 391–394 (1966).
R, P	253. Polyanskii, V. K., and Rvachev, V. P., Light reflection from rough surfaces, Report No. RSIC-588 (Opt. Spectrosc. **20** (4), 701–708, 1966) AD-641 140.
R, Th	254. Porteus, J. O., Relation between the height distribution of a rough surface and the reflectance at normal incidence, J. Opt, Soc. Am. **53** (12), 1394–1402 (Dec. 1963).
SM	255. Posey, C. J., Measurement of surface roughness, Mech. Eng. **68** (4), 305–306, 338 (April 1966).
R, Ex, Th	256. Pouey, M., and Fabre, D., Quelques experiences de diffusion du rayonnement ultraviolet par des surfaces rugueuses, Optica Acta **16** (4), 471–481, 1969.
SM	257. Reason, R. E., The application of an electronic computer to the study of surface measurement, Automatisme **9** (5), (May 1964).
SM	258. Reason, R. E., Workshop requirements of surface measurement, pp. 299–305, PMS* Proceedings 1967–68.
R, S, Ex(L)	259. Renau, J. and Collinson, J. A., Measurements of electromagnetic backscattering from known, rough surfaces, The Bell Syst. Tech. Journal, **44** (10), 2203–2226 (Dec. 1965).
S, P, Ex	260. Renau, J., Cheo, P. K., and Cooper, H. G., Depolarization of linearly polarized EM waves backscattered from rough metals and inhomogeneous dieleetrics, J. Opt. Soc. Am. **57** (4), 459–466 (1967).
R, P, Th, Ex	261. Rense, W. A., Polarization studies of light diffusely reflected from ground and etched glass surfaces, J. Opt. Soc. Am. **40**, 55–59 (1950).
SM	262. Ribbens, W. B., Interferometric surface roughness measurement, Appl. Opt. **8** (11), 2173–2176 (1969).
Ma	263. Rice, S., Mathematical analysis of random noise, I & II, Bell Sys. Tech. J. **23**, 282–332 (1944); III & IV, Bell Sys. Tech. J. **24**, 45–156 (1945).
R, Th	264. Rice, S. O., Reflection of electromagnetic waves from slightly rough surfaces, Comm. Pure Appl. Math. **4**, 351–378 (1951).
SM	265. Richards, P.J., Review of methods of measurement and assessment of surface form and texture, pp. 453–465, PMS* Proceedings 1967–68.
R, Th	266. Richmond, J. C., Effect of surface roughness on emittance of nonmetals, AIAA Thermophysics Specialist Conference, Monterey, Calif., AIAA Paper No. 65-675 (Sept. 1965). J. Opt. Soc. Am. **56** (2), 253–254 (Feb. 1966).
SM	267. Richmond, J. C. and Francisco, A. C., Use of plastic replicas in evaluating surface texture of enamels, J. Res. Nat. Bur. Stand. (U.S.), **42**, 449–460 (May 1949). RP1985.
R, Th	268. Rolling, R. E., The effect of slight surface roughness on emittance, NASA SP-55, Symposium on Thermal Radiation of solids (S. Katzoff, ed.), pp. 153–155, NASA (1965).
Em, Ex	269. Rolling, R. E., Effect of surface roughness on the spectral and total emittance of platinum, AIAA Paper No. 67-320, AIAA Thermophysics Specialist Conference, New Orleans, La. (Apr. 1967).
R, Ex, Th	270. Rolling, R. E. and Funai, A. I., Investigation of the effect of surface condition on the emittance and reflectance of opaque materials, Quarterly Progress Reports, 1963, Contract No. AF 33 (657)-11281, task No. 736001, Aerospace Science Laboratory, Lockheed Missiles and Space Co., Palo Alto, Calif., 1963.
Em, P, Ex	271. Rolling, R. E. and Funai, A. I., Investigation of the effect of surface conditions on the radiant properties of metals, Part II, Measurements on roughened platinum and oxidized stainless steel, AFML-TR-64-363 (Apr. 1964).
S, Th	272. Ross, R. A., Small-angle scattering by a finite cone, IEEE Trans., on Antennas and Propagation **AP-17** (2), 241–242 (1969).
R, Ex	273. Rouse, J. W., Jr., Wavelength dependence of backscatter from rough surface, Tech. Rept. RSC-12, Remote Sensing Center, Texas A. & M. Univ., 1–21 (Sept. 1970).
S, Th	274. Rousseau, M., Statistical properties of optical fields scattered by random media. Application to rotating ground glass, J. Opt. Soc. Am. **61** (10), 1307–1316 (Oct. 1971).
S, H	275. Roux, J. A., Radiative heat transfer of coatings on a cryogenic surface, AEDC-TR-71-90, 1-205, Von Karman Gas Dynamics Facility, Arnold Engineering Development Center, Air Force Systems Command, Arnold Air Force Station, Tenn. (Apr. 1971).
R, Ex	276. Russell, D. A., The spectral reflectance of rough surfaces in the infrared, Master’s thesis, Dept. of Mechanical Engineering, Univ. of Calif. at Berkeley (1961).
SM	277. Sadowski, A., Trends and actual state of Polish scientific research work in the field of surface metrology, pp. 229–240, PMS* Proceedings, 1967–68.
R, Ex, Th	278. Safwat, H. H. and Parmer, J. F., Effect of surface roughness on specular and diffuse reflectance components of carbon steel in visible wavelength range, ASME Paper No. 69-WA/HT-42, ASME Winter Annual Meeting, Nov. 1969.
S, Th	279. Sancer, M. I., Shadow-corrected electromagnetic scattering from a randomly rough surface, IEEE Trans. on Antennas and Propagation **AP-17** (5), 557–585 (1969).
R, Th	280. Schmidt, R. N., Infrared diffuse reflector coating, Honeywell Doc. 12041-QR2, 1967.
SM	281. Schneider, E. J., Surface finish testing. The present state of the art and its future, Engis Equipment Co. Morton Grove, 111. (Sept. 15, 1969).
Em, Th	282. Schocken, K., The angular distribution of infrared radiation, Rept. No. DV-TN-3-60, Research Projects Laboratory, George C. Marshall Space Flight Center (Feb. 1960).
R(L), Ex, Ap	283. Seaney, R. J., The angular backscattering characteristics of plane surfaces illuminated by carbon dioxide laser radiation, R.A.R.D.E. Memorandum 3/69 Royal Armament Research and Development Establishment (Feb. 1969). AD-850 443.
S, Th	284. Senior, T.B.A., The scattering of electromagnetic waves by a corrugated sheet, Can. J. Phys. **37** (7), 787–797, 1572 (1959).
S, Th, Ex	285. Senior, T. B. A., Surface roughness and tolerances in model scattering experiments, IEEE Trans. on Antennas and Propagation **AP-13** (4), 620–636 (1965).
SM	286. Sharman, H. B., Influence of sample size and the relationships between the common surface texture parameters, pp. 416–424 PMS* Proceedings 1967–68.
R, Th	287. Shaw, L., Comments on ‘Shadowing of random surfaces’, IEEE Trans. on Antennas and Propagation (Communications) **AP-14**, 253 (Mar. 1966).
R, Ex	288. Shelley, R., Sea surface forward scattering at low grazing angles, Navy Electronics Lab., San Diego Calif., Rept. No. NEL-1338, 1–30 (Nov. 1965). AD-480 126.
S, Th	289. Shubert, H. A., Plane wave diffraction by thick dielectric gratings and zone phase plates, Tech. Rept. No. 77, 1-149, Illinois Univ. Urbana Engineering Experiment Station (June 1964). AD-603 026.
Em, Ex	290. Siegfried, R. G. and Birkebak, R. D., A comparative study of the total directional emittance of a dielectric and a metal as a function of surface roughness, AIAA 3rd Thermophysics Conference, Los Angeles, Calif., AIAA Paper No. 68-778 (June 1968).
R, Th	291. Simmons, E. L., An equation relating the diffuse reflectance of weakly absorbing powdered sample to the fundamental optical parameters, Optica Acta **18** (1), 59–68 (1971).
P, Th	292. Sirohi, R. S., Optical constants of rough surface by ellipsometry, Brit. J. Appl. Phys. **3** (9), 1407–1409 (1970).
S, Th	293. Sledge, O. D., The scattering of a plane electromagnetic wave by a linear array of center loaded cylinders, Rept. No. NRL-6681, 1–45, Naval Research Lab., Wash. D.C. (June 1968). AD-672 182.
S, R, Th	294. Sledge, O. D., Scattering of a plane electromagnetic wave by a linear array of cylindrical elements, IEEE Trans. on Antennas and Propagation **AP-17** (2), 169–174 (1969).
R, Ex	295. Smith, A. M. and Muller, P. R., Super- and sub-specular maxima in the angular distribution of polarized radiation reflected from roughened dielectric surfaces, AEDC-TR-70-286, Von Karman Gas Dynamics Facility, Arnold Engineering Development Center, Air Force Systems Command, Arnold Air Force Station, Tenn. (1971).
T (Ex, Th)	296. Smith, A. M. and Muller, P. R., Anomalous refraction maxima in the bidirectional transmittances of roughened dielectric surfaces, AIAA Paper No. 72-302, 1–11, AIAA 7th Thermophysics Conference, San Antonio, Tex. Apr. 10–12, 1972.
R, Ex	297. Smith, A. M., Muller, P. R., Frost, W., and Hsia, Han M., Super- and sub-specular maxima in the angular distribution of polarized radiation reflected from roughened dielectric surfaces, AIAA Paper No. 70-861, AIAA 5th Thermophysics Conference, June 1970.
S, Th	298. Smith, B. G., Geometrical shadowing of a random rough surface, IEEE Trans. Antennas and Propagation **AP-15**, 668–671 (Sept. 1967).
R, Th	299. Smith, T. F., and Hering, R. G., Bidirectional reflectance of a randomly rough surface, AIAA Paper No. 71-465, AIAA 6th Thermophysics Conference, Tullahoma, Tenn., Apr. 26–28, 1971.
R, Su, Th, Ex, Ap	300. Smith, T. F., and Hering, R. G., Surface roughness effects on bidirectional reflectance, Tech. Rept. No. ME-TR-661-2, Heat Transfer Laboratory, University of Illinois, Urbana, Ill. (June 1972).
R, Th, Ex	301. Solesvik, A., Effective reflectivity and angular distribution of light reflected from sea water with surface waves, Norwegian Defense Research Establishment, Rept. No. Teknisk notat E-118 (Sept. 1966). AD-815 675.
R, Ex, Su	302. Sparrow, E. M., and Cess, R. D., Radiation Heat Transfer, pp. 60–63 (Wadsworth Publishing Co., Belmont, Calif., 1966).
R, Th	303. Spencer, D. E., and Gaston, E. A., The numerical ranges of diffuse and specular reflectance, Dept. of Mathematics U-9, The University of Connecticut, Storrs, Conn. (1973). Unpublished report.
R, Th	304. Spetner, L. M., Discussion of H. Davies’ The reflection of electromagnetic waves from a rough surface, Proc. IEE, Pt. III, **102**, 148 (1955).
R, S, Th	306. Spetner, L. M., A statistical model for forward scattering of waves off a rough surface, IRE Trans, on Antennas and Propagation **AP-6** (1), 88–94 (Jan. 1958).
S, Th, Ex	307. Sporton, T. M., The scattering of coherent light from a rough surface, Brit. J. Appl. Phys. (J. Phys. D), Ser. 2, **2** (7), 1027–1034 (1969).
SM	308. Spragg, R. C, Accurate calibration of surface texture and roundness measuring instruments, pp. 397–405, PMS* Proceeding, 1967–68.
Ex, R	309. Stanford, J. L., Ashley, E. J., and Bennett, H. E., Roughness-induced absorption in metallic mirrors, Preprint of a paper presented at the Optical Society of America 1970 Annual Meeting, Sept. 28-Oct. 2, Held at Hollywood, Fla.
R, Ex	310. Stevison, D. F., Effect of surface roughness on the reflectance of refractory metals, AFML-TR-66-232 (Dec. 1966).
S, Th	311. Stogryn, A., Electromagnetic scattering from rough, finitely conducting surfaces, Radio Sci. **2**, 415–428 (1967).
SM	312. Strang, A. G., and Ogborn, F., Measurement of surface roughness of electrodeposited and electropolished surfaces by means of the micro-interferometer, NBS Handbook 77, Vol. III: Precision Measurement and Calibration-Optics, Metrology and Radiation, 324/1-335/12 (1961).
S, Th	313. Swift, C. T., A note on scattering from a slightly rough surface, IEEE Tran, on Antennas and Propagation **AP-19** (4), 561–562 (July 1971).
H	314. Toor, J. S. and Viskanta, R., Experiment and Analysis of directional effects on radiant heat transfer, J. Heat Transfer, 459–466 (Nov. 1972).
H	315. Toor, J. S., Viskanta, R., and Winter, E. R. F., An experimental and analytical study of real surface effects on radiant heat transfer, Heat Transfer Laboratory Rept., Mechanical Engineering School, Purdue University (May 1971).
R, Ex	316. Toporets, A. S., Specular reflection from a rough surface, Opt. Spectrosc. **16**, 54–58 (1964).
P, Ex	317. Toporets, A. S. and Mazurenko, M. M., Depolarization of light upon diffuse reflection, Soviet J. Opt. Tech. **35** (1), 81–83 (1968).
R, P, Ex	318. Toporets, A. S., Specular reflection of polarized light from rough surfaces, Soviet J. Opt. Technology **35** (4), 419–422 (1968).
R, Ex	319. Toporets, A. S., Specular reflection of light from a rough surface, Opt. Spectrosc. **24** (1), 62–64 (Jan. 1968).
R, Th, Ex	320. Torrance, K. E., Off-specular peaks and angular distribution of reflected thermal radiation, Ph. D. dissertation, University of Minnesota, Minneapolis, Minn., March 1966.
R, P, Th	321. Torrance, K. E., Theoretical polarization of off-specular reflection peaks, J. Heat Transf., Trans. ASME **C91** (2), 287–290 (1969).
R, Ex	322. Torrance, K. E. and Sparrow, E. M., Discussion of Effects of roughness of metal surfaces on angular distribution of monochromatic radiation, J. Heat Trans. ASME **C87**, 93 (1965).
R, Ex	323. Torrance, K. E., and Sparrow, E. M., Biangular reflectance of an electric nonconductor as a function of wavelength and surface roughness, J. Heat Transf., Trans. ASME **C87**, 283–291 (1965).
R, Ex	324. Torrance, K. E. and Sparrow, E. M., Off-specular peaks in the directional distribution of reflected thermal radiation, J. Heat Transf. Trans. ASME **C88**, 223–230 (1966).
R, P, Ex	325. Torrance, K. E., Sparrow, E. M., and Birkebak, R. C., Polarization, directional distribution, and off-specular peak phenomena in light reflected from roughened surfaces, J. Opt. Soc. Am. **56** (7), 916–925 (1966).
R, Th	326. Torrance, K. E. and Sparrow, E. M., Theory for off-specular reflection from roughened surfaces, J. Opt. Soc. Am. **57** (9), 1105–1114 (1967).
H, Th	327. Treat, C. H., Directional effects in radiant heat transfer between pairs of simply arranged surfaces, Ph. D. Dessertation, Univ. of New Mexico, Albuquerque, N. Mex., 1-154 (Jan. 1968).
R, Th	328. Treat, C. H. and Wildin, M. W., Investigation of a model for bidirectional reflectance of rough surfaces, AIAA Paper No. 69-64, AIAA 7th Aerospace Sciences Meeting, New York City, N.Y., Jan. 20–22, 1969.
R, Ex	329. Trotter, A. P., Diffused reflection and transmission of light, Illuminating Engineer **12**, 243–267 (Sept. 1919).
R, Th	330. Trowbridge, T. S. and Reitz, K. P., New ray optics model for the directional distribution of light reflected from a rough surface, Naval Missile Center, Point Mugu, Calif. (1973). Unpublished report.
SM	331. Trumpold, H., Limits of application of the interference methods for surface measurements, pp. 241–254, Proceedings PMS * 1967–68.
R, Th	332. Twersky, V., On the nonspecular reflection of electromagnetic waves, J. Appl. Phys. **22** (6), 825–835 (Jan. 1951).
S, Th	333. Twersky, V., Multiple scattering of radiation by an arbitrary planar configuation of parallel cylinders and by two parallel cylinders, J. Appl. Phys. **23** (4), 407–414 (1952).
R, S, Th	334. Twersky, V., Reflection coefficients for certain rough surfaces, J. Appl. Phys. **24** (5), 659–660 (1953).
S, Th	335. Twersky, V., Scattering theorems for bounded periodic structures, J. Appl. Phys. **57** (10), 1118–1122 (Oct. 1956).
S, Th	336. Twersky, V., On the scattering of waves by an infinite grating, IRE Trans. on Antennas and Propagation **AP-4** (3), 330–345 (July 1956).
R, S, Th	337. Twersky, V., On scattering and reflection of electromagnetic waves by rough surfaces, IRE Trans. on Antennas and Propagation **AP-5** (1), 81–90 (Jan. 1957).
S, Th	338. Twersky, V., Scattering by quasiperiodic and quasirandom distributions, IRE Trans. on Antennas and Propagation **AP-7** (Special Supplement) 307–319 (Dec. 1959).
S, Th	339. Twersky, V., Multiple scattering of waves and optical phenomena, J. Opt. Soc. Am. **52** (2), 145–171 (Feb. 1962).
S, Th	340. Twersky, V., On scattering of waves by the infinite grating of circular cylinders, IRE Trans. on Antennas and Propagation **AP-10** (6), 737–765 (Nov. 1962).
Su	341. Ulrich, J., Unusual reconnaissance concepts Vol. **III:** A bibliography of recent contributions on electromagnetic and acoustic scattering, Tech. Rept. AFAL-TR-65-331, Vol. **III**, Willow Run Lab., Institute of Science and Technology, University of Michigan, Ann Arbor, Mich. (Jan. 1966).
R, Th	342. Valenzuela, G. R., The effective reflection coefficients in forward scatter from a dielectric slightly rough surface, Proc. IEEE (U.S.) **58** (8), 1279 (1970).
S, Th	343. Venable, W. H., Jr., Effects upon radiant intensity measurements due to scattering by optical elements, Appl. Opt. **9** (3), 609–615 (Mar. 1970).
R, Th	344. Vincent, R. K., and Hunt, G. R., Infrared reflectance from mat surfaces, Appl. Opt. **7** (1), 53–59 (Jan. 1968).
H, Th, Ex	345. Viskanta, R., Schornhorst, J. R., and Toor, J. S., Analysis and experiment of radiant heat exchange between simply arranged surfaces, Tech. Note May 65-June 67, 1–131, Purdue Univ. Lafayette Indiana, School of Mechanical Engineering (June 1967). AD-655 335.
R, Ex	346. Voishvillo, N. A., Reflection of light by a rough glass surface at large angles of incidence of the illuminating beam, Opt. Spectrosc. **22** (6), 517–520 (June 1967).
SM	347. Von Weingraber, H., Optical methods for surface finish evaluation, International Research in Production Engineering, pp. 659–666 (1963). Published by ASME.
R, S, Th	348. Wagner, R., Shadowing of randomly rough surfaces, J. Acoust. Soc. Am. **41** (1), 138–147 (1967).
S, Th	349. Wait, J. R., Reflection at arbitrary incidence from a parallel wire grid, Appl. Sci. Res. **B4**, 393–400 (1954–55).
S, Th	350. Wait, J. R., On the theory of reflection from a wire grid parallel to an interface between homogeneous media (I), Appl. Sci. Res. **B6**, 259–275 (1957).
S, Th	351. Wait, J. R., On the theory of reflection from a wire grid parallel to an interface between homogeneous media (II), Appl. Sci. Res. **B7**, 355–360 (1958–59).
R, Th	352. Wait, J. R., Guiding of electromagnetic waves by uniformly rough surfaces Part I & II, IRE Trans. on Antennas and Propagation **AP-7** (Special Supplement), 154–168 (Dec. 1959).
S, Th	353. Wait, J. R., Pertubation analysis for reflection from two-dimensional periodic sea-waves, Radio Sci. **6** (3), 387–391 (1971).
R(Ex)	354. Walter, F. E. and Wiebelt, J. A., An investigation of bi-directional reflectance from randomly rough engineering surfaces, AIAA Paper No. 72-308, 1–9, AIAA 7th Thermophysics Conference, San Antonio, Tex., Apr. 10–12, 1972.
S, Th, Ex	355. Waterman, P. C., Matrix formulation of bistatic electromagnetic scattering, Rept. No. MTR-543, ESD-TR-67-629, 1–26, Mitre Corp. Bedford, Mass. (May 1968). AD-669 093.
S, Th	356. Waterman, P. C. and McCarthy, C. V., Numerical solution of electromagnetic scattering problems, Rept. No. MTP-74, 1–132, Mitre Corp. Bedford, Mass. (June 1968). AD-685 715.
R, Ex	357. Weaver, J. H., Recent advances in optical properties of oxide and intermetallic coatings, AFML-TR-65-29, 220–235 (1965).
S, Th	358. Weston, V. H., Boerner, W. M., and Hodgins, D. R., Inverse scattering investigation, Quarterly Rept. No. ESD-TR-67-517, Dept. of Electrical Engineering, Radiation Lab., Univ. of Michigan, 1968.
SM	359. White, E. W., McKinstry, H. A., and Diness, A., Quantitative surface finish characterization by CESEMI, Preprint of paper presented at the Symposium on the Science of Ceramic Machining and Surface Finishing, Nov. 2–4, 1970. Held at the National Bureau of Standards, Gaithersburg, Md.
SM	360. White, E. W., McKinstry, H. A. and Diness, A., Quantitative surface finish characterization by CESEMI, Tech. Rept. No. 4, Office of Naval Research, Metallyrgy Program; Material Research Lab. The Pennsylvania State University, University Park, Pa. (1971). AD-719 925.
SM	361. Whitehouse, D. J., and Reason, R. E., The equation of the mean line of surface texture found by an electric wave filter, Rank Taylor Hobson, Leicester, Great Britain (1965).
SM	362. Whitehouse, D. J., Improved type of wavefilter for use in surface-finish measurement, pp. 306–318, PMS* Proceedings, 1967–68.
SM	363. Whitehouse, D. J., Discussion, pp. 498–502, PMS* Proceedings, 1967–68.
SM	364. Williamson, J. B. P., Microtopography of surfaces, pp. 21–30, PMS* Proceedings, 1967–68.
SM	365. Wolff, H., Surface testing using the Hommel tester type T, pp. 279–298, PMS* Proceedings, 1967–68.
S, Th	366. Yen, J. L., Multiple scattering and wave propagation in periodic structures, IRE Trans. on Antennas and Propagation **AP-10** (6), 769–775 (Nov. 1962).
R, Th	367. Zaki, K. A., and Neureuther, A. R., Scattering from a perfectly conducting surface with a sinusoidal height profile: TE polarization, IEEE Trans. on Antennas and Propagation **AP-19** (2), 208–214 (1971).
R, Th	368. Zhitomirskii, I. S., Chebanova, R. S., Shakhnovich, M. I., Effect of self-shadowing on the coefficient of reflection from the cleaved surfaces of a monocrystal, Opt. Spectrosc. **19** (3), 229–232 (Sept. 1965).
Em, Th	369. Zhulev, Yu. G., Radiative power of a toothed surface, (Translated from) Teplofizika Vysokikh Temperatur **3** (2), 321–323 (Mar.–Apr. 1965).
R, Th, Ex	370. Zipin, R. B., The apparent thermal radiation properties of an isothermal V-groove with specularly reflecting walls, J. Res. Nat. Bur. Stand. (U.S.), **70C** (Eng. *&* Instr.), No. 4, 275–280 (Oct–Dec. 1966).
R, Th, Ex	371. Zipin, R. B., A preliminary investigation of the bidirectional spectral reflectance of V-grooved surfaces, Appl. Opt. **5** (12), 1954–1957 (1966).

* Properties and Metrology of Surface–Proceedings 1967–68, Vol. **82**, part 3k, Presented at International Conference organized by the Institution of Mechanical Engineers at Oxford, 1st to 4th April 1968.

